# Navigating Complexity: A Comprehensive Approach to Middle Cerebral Artery Aneurysms

**DOI:** 10.3390/jcm13051286

**Published:** 2024-02-24

**Authors:** Anna Maria Auricchio, Rina Di Bonaventura, Enrico Marchese, Giuseppe Maria Della Pepa, Carmelo Lucio Sturiale, Grazia Menna, Benjamin Skrap, Alessandro Olivi, Alessio Albanese

**Affiliations:** 1 Department of Neurosurgery, Fondazione Policlinico Universitario A. Gemelli IRCCS, Università Cattolica del Sacro Cuore, 00168 Rome, Italy; anna.maria90a@gmail.com (A.M.A.); rina.di.bonaventura@hotmail.it (R.D.B.); enrico.marchese@unicatt.it (E.M.); giuseppemaria.dellapepa@policlinicogemelli.it (G.M.D.P.); benjamin.skrap@gmail.com (B.S.); alessandro.olivi@policlinicogemelli.it (A.O.); 2 Department of Neurosurgery, UMC Utrecht, 3584 CX Utrecht, The Netherlands

**Keywords:** middle cerebral artery, intracranial aneurysms, unruptured, complex aneurysms, surgical clipping, endovascular treatment, complexity criteria, treatment algorithm

## Abstract

**Background:** The concept of aneurysm “complexity” has undergone significant changes in recent years, with advancements in endovascular treatments. However, surgical clipping remains a relevant option for middle cerebral artery (MCA) aneurysms. Hence, the classical criteria used to define surgically complex MCA aneurysms require updating. Our objective is to review our institutional series, considering the impacts of various complexity features, and provide a treatment strategy algorithm. **Methods:** We conducted a retrospective review of our institutional experience with “complex MCA” aneurysms and analyzed single aneurysmal-related factors influencing treatment decisions. **Results:** We identified 14 complex cases, each exhibiting at least two complexity criteria, including fusiform shape (57%), large size (35%), giant size (21%), vessel branching from the sac (50%), intrasaccular thrombi (35%), and previous clipping/coiling (14%). In 92% of cases, the aneurysm had a wide neck, and 28% exhibited tortuosity or stenosis of proximal vessels. **Conclusions:** The optimal management of complex MCA aneurysms depends on a decision-making algorithm that considers various complexity criteria. In a modern medical setting, this process helps clarify the choice of treatment strategy, which should be tailored to factors such as aneurysm morphology and patient characteristics, including a combination of endovascular and surgical techniques.

## 1. Introduction

The concept of aneurysm “complexity” has evolved significantly in recent years due to advancements in endovascular treatments. What was once considered challenging for surgical clipping can now often be successfully managed through endovascular techniques, and vice versa [1]. While the exact global incidence of complex aneurysms remains unclear [2], they continue to pose a significant challenge for both neurosurgeons and interventional radiologists. Several complexity criteria have been reported in the literature, including fusiform shape, large or giant size, vessel origin from the sac, thrombosis, previous treatments, wide neck, proximal vessel tortuosity, and calcifications [3]. The unruptured intracranial aneurysm score (UIATS) defines the aneurysmal complexity given the presence of a neck wider than the diameter of the parent vessel, calcified dome or proximal vessel tortuosity/stenosis, aneurysm thrombosis, or sac smaller than 3 mm [3,4,5]. Giant aneurysms and cases with a previous endovascular or surgical treatment can be likewise considered complex [4,6,7,8]. The Berlin classification emphasizes this concept in MCA aneurysms, driving best management practices in thrombosed aneurysms [4]. Any combination of these features can render conventional clipping unfeasible. While new definitions and classifications of aneurysm complexity have emerged [1,3,5], they often lack a quantitative assessment of the combined qualitative features and their relevance for treatment. This correlation between complexity features and treatment is increasingly crucial in a multidisciplinary context, especially for middle cerebral artery (MCA) aneurysms, which are the most frequently surgically treated [9,10,11,12,13,14]. The correct identification of complexity features in MCA aneurysms is fundamental in preoperatively identifying the possible pitfalls during the treatment.

Arising from the internal carotid artery, the MCA branches into two main divisions, the M1 and M2 segments. The M1 segment travels through the Sylvian fissure, giving rise to several branches that supply regions of the brain relevant for motor and speech functions. In more detail, the MCA is structured in the proximal (M1) and bifurcational and distal segments (M2–M5), originating at the anterior perforate substance, where the carotid terminus leaves the anterior cerebral artery and the MCA. The MCA gracefully traces alongside the sphenoid ridge before executing a slight 90° turn at the limen insula. As it progresses, the MCA’s M2 and M3 segments find themselves nestled within the Sylvian cistern, ultimately branching out onto the frontoparietal cortex as M4 and M5. The bifurcation of the MCA, typically found located between the frontal and temporal opercula, maintains a posterior connection with the limen insula and an anterior alignment with the sphenoid ridge. The MCA bifurcation frequently predates the genu of the insula and can host lenticulostriate arteries, which must be delicately identified and avoided during aneurysm clipping procedures. The superficial portion of the Sylvian fissure splits in the anterior horizontal, anterior ascending, and posterior ramus [15], finding an important cortical landmark for the MCA in the Sylvian cistern in the pars opercularis of the frontal lobe. However, it is essential to recognize that the Sylvian fissure’s characteristics can vary significantly, encompassing differences in depth, width, length, venous size, and the spatial relationship between the frontal and temporal lobes. Understanding these nuances unlocks a deeper appreciation for the intricate topography of the brain’s lateral surface and aids in navigating the delicate intricacies of neurosurgical procedures such as the surgical treatment of complex MCA aneurysms.

The complexity of the middle cerebral artery’s branching pattern, combined with variations in the sizes and shapes of aneurysms, underscores the need for a nuanced approach to its surgical management.

In this paper, we aim to achieve the following objectives:

(1) Review our institutional series of complex MCA aneurysms, assessing the impacts of complexity features on the decision-making process for surgical strategy;
(2) Provide a treatment strategy algorithm based on different complexity “portraits”.

## 2. Materials and Methods

### 2.1. Analysis of the Institutional Series

We conducted a retrospective review of all unruptured MCA aneurysms admitted to our institution between August 2018 and January 2023. We examined clinical and surgical reports as well as radiological imaging. Our series included surgically treated complex cases selected based on complexity criteria from the previous literature [2,4,13,16,17,18,19,20,21,22], such as the UIATS and Berlin classification [3,4,23]. We distinguished “major” and “minor” criteria of complexity: major features were those able to significantly influence the decision-making process for surgery. We defined a complex MCA aneurysm if it met at least one “major” criterion (e.g., fusiform shape, large or giant size, vessel originating from the aneurysmal dome, thrombosis, and previous treatment) or a combination of “major” and “minor” complexity criteria (e.g., wide neck, proximal vessel tortuosity, and calcification). We analyzed demographic data, risk factors for vasculopathy, clinical presentation, and preoperative modified Rankin score (mRS). Radiological assessments were performed with Computed Tomography Angiography (CTA) or Digital Subtraction Angiography (DSA). All procedures were assisted by Intraoperative Neuromonitoring and Indocyanine Green Videoangiography. Surgical strategies included complex clipping reconstruction, trapping, and bypass. The preservation of cerebral distal flow and parent vessel patency and the complete exclusion of the aneurysm sac were assessed by postoperative radiological imaging. Within our institution, all the vascular cases were discussed in the multidisciplinary neurovascular board.

### 2.2. Statistical Analysis

Quantitative variables were expressed as mean ± standard deviation. Comparison of continuous variables between groups was performed using the Mann–Whitney U test, while categorical variables were compared using the Chi-square statistic. Statistical significance was set at *p* ≤ 0.05. Analyses were conducted using SPSS v25^®^.

## 3. Results

### 3.1. Institutional Population of MCA Complex Cases

In our institutional series of 188 surgically treated unruptured MCA aneurysms, we identified 14 cases (7%) meeting the criteria for complexity.

The distribution of complexity features is as follows:

- Fusiform shape: 57%.
- Large size: 35%.
- Giant size: 21%.
- Vessel originating from the sac: 50%.
- Intraluminal thrombi: 35%.
- Previous treatment (clipping or coiling): 14%.

Notably, 92% of these complex cases had a wide neck, and 28% exhibited proximal vessel tortuosity or stenosis. Furthermore, wall calcification was present in 14% of aneurysms, contributing to their complexity. At least two complexity criteria coexisted in all the aneurysms of our series (Table 1).

### 3.2. Demographics and Clinical Presentation

The mean age of patients with complex MCA aneurysms was 54.85 ± 16.18 years. The majority were female (64%). Risk factors for vasculopathy, such as hypertension, smoking, and hypercholesterolemia, were present in 71% of cases. Clinical presentation varied, with the most common symptoms being headache (71%), followed by seizures (21%). The preoperative mRS score was 0–2 in 78% of cases (Table 2).

### 3.3. Radiological Findings

Radiological assessments showed that complex MCA aneurysms were located predominantly in the M2 segments (35%) of the MCA. The mean aneurysm size was 14.36 ± 8.7 mm (Table 3) One-third of the aneurysms were partially thrombosed (35%) and had a wide neck (92%). Tortuosity or stenosis of proximal vessels was noted in 28% of cases. Furthermore, in 14% of cases there was wall calcification.

### 3.4. Surgical Strategies

Surgical strategies for complex MCA aneurysms were tailored to individual cases. The following approaches were employed (Table 4):

- Complex clipping reconstruction: 64%.
- Aneurysm trapping and by-pass: 36%.

In complex clipping reconstruction, various techniques were used, including wrapping. Aneurysm trapping was performed in cases where the complexity features precluded safe clipping. Bypass procedures were conducted when necessary to maintain blood flow.

### 3.5. Postoperative Outcomes

Postoperative outcomes were generally favorable. Immediate angiographic results showed complete obliteration in 86% of cases. Postoperatively, all patients experienced clinical improvement, with 93% of patients with 0–2 mRS at discharge. Complications were infrequent and included one case of transient focal neurological deficit and one case of postoperative hydrocephalus (Table 3).

## 4. Discussion

Unruptured intracranial aneurysms are vascular lesions whose incidence is continuously increasing in the global population due to incidental radiological findings [3]. Endovascular strategy has been demonstrated to be the first choice for posterior circulation, both for ruptured and unruptured aneurysms [1,24,25]. More frequently, in the anterior circulation, the new endovascular devices are especially used for anterior communicating complex and proximal anterior cerebral artery aneurysms [26,27]. Despite these advancements in the endovascular era, MCA aneurysms are still mainly treated surgically by traditional clipping, with safety and effectiveness, in high-volume centers [1,28]. On the other hand, new endovascular techniques have also made possible treatments specific to MCA aneurysms [26]. This concept has demonstrated that aneurysms surgically treated are often the most challenging and complex. In recent decades, the surgical techniques have been progressively improved, with the miniaturization of the approaches, the focal opening of the Sylvian fissure, and the utilization of the retractorless technique [29,30]. The shortening of hospitalization and the decrease of intracranial complications (e.g., contusions, hematomas, and seizures) have obtained excellent results, combined with the complete exclusion of the malformation. However, the management of complex MCA aneurysms remains a challenge due to the evolving concept of aneurysm complexity and the interplay of different factors influencing treatment decisions. The need for a tailored approach is paramount in effectively addressing these intricate cases [29,30,31]. We analyzed the complexity features in our series of unruptured intracranial MCA aneurysms and proposed an algorithm for treatment as described in the following.

### 4.1. Importance of Major Criteria in Complexity Profile

The analysis of preoperative mRS in unruptured complex MCA aneurysm cases documented that 22% of patients were symptomatic, which was unlike the corresponding percentage for patients with non-complex unruptured MCA aneurysms. This result corroborates the need for treatment of these complex cases. Furthermore, among our series of unruptured complex MCA aneurysms, we observed that the trends of age, female/male ratio, postoperative mRS, and radiological outcome showed no difference between the general population of unruptured non-complex MCA aneurysms and the cases of complex MCA aneurysms. This result suggests that the good outcome is basically fostered by proper multidisciplinary discussion of each case during the vascular board and the subsequent successful management. In fact, the surgical strategy was directed, prioritizing the presence of major criteria such as fusiform shape, large/giant size, vessel branching from the dome, thrombosis, and previous treatment. The mean size of the complex MCA aneurysm series was higher than that of the unruptured non-complex MCA population with a significant result (*p* > 0.00001). This aspect endorses the inclusion of wide size aneurysms, as usual, in a complexity profile. Other criteria of complexity, such as wide neck, wall calcification, and proximal vessel stenosis/tortuosity, were considered minor features. Hence, the major criteria were resultingly of paramount importance for the decision-making process opting between complex clipping reconstruction or partial/complete trapping and bypass.

### 4.2. Algorithm for Treatment Strategy

(1) The **Preoperative Assessment** is based on the evaluation of the aneurysm’s morphology, including size, shape, presence of thrombosis, neck width, and vessel tortuosity or stenosis, and the analysis of patient demographics, clinical presentation, and risk factors for vasculopathy. The multidisciplinary discussion of each vascular case among neurointerventional radiologists and neurosurgeons represents an approach essential for optimizing patient care.
(2) The **Identification of the Specific Complexity Profile** [2,19,30] is determined by the combination of major and minor complexity criteria. Major criteria include fusiform shape, large/giant size, vessel originating from the sac, intrasaccular thrombi [3], and previous treatments [6,7,8]. Minor criteria encompass wide neck, proximal vessel tortuosity, and wall calcification.
(3) The **Treatment Decision** is tailored based on the preoperative assessment and complexity profile. Despite the fact that the complex clipping reconstruction technique appears to be the most common strategy used in our series, major criteria significantly orientated the choice of treatment. Among them, fusiform shape was the one that mainly drew us towards choosing bypass as a rescue strategy in cases of unfeasible clipping reconstruction. Indeed if distal flow could not be preserved with the clipping strategy, trapping and bypass were required [17,32,33].
(4) The **Intraoperative setting** for complex aneurysm cases benefits from the combination of flow-preservation tools with Microdoppler flowmetry, Indocyanine Green Videoangiography, and continuous Intraoperative Neuromonitoring [34,35]. The standard neuromonitoring protocol, including motor-evoked potentials and somatosensory-evoked potentials of the contralateral side, as well as EEG, permits increased efficiency and safety of the complex aneurysm exclusion and bypass creation [31,36].

### 4.3. Complexity Portraits and Surgical Treatment Algorithm

A surgical-strategy decision making process deals with analysis of the “complexity portrait” of the single aneurysm and how it changes the steps of surgical management: proximal control, sac dissection, parent vessel dissection and preservation, temporary clipping, and aneurysm exclusion. One or more of these steps may be difficult or impossible to achieve and the aneurysm exclusion can then only be obtained by vessel sacrifice preceded by distal revascularization.

Lawton, in 2017, reported his experience with complex MCA aneurysms, and described surgical revascularization as the best treatment option [17]. On the other hand Esposito et al., in 2014, proposed different approaches, including reconstruction with eventual protective bypass, and complete or partial trapping with revascularization [19]. Reviewing our series, we found out that we could obtain excellent clinical and radiological outcomes through a strategy which considers, as the first option, the reconstruction of the aneurysmatic lesion. In our experience, trapping and vessel sacrifice after flow replacement may be considered a rescue strategy. We assume the first option to be the “gross total” reconstruction, which eventually leaves a low-risk bleeding remnant. Nevertheless, there is no evidence of a risk of bleeding from “gross total” reconstructed aneurysms [33]. The choice between “gross total” reconstruction and vessel sacrifice with revascularization mainly depends on the patient’s age and the aneurysm’s previous bleeding, size, morphology, and eventual suitability for endovascular treatment of the remnant.

In our series, the complexity criteria influencing the decision-making process were fusiform shape, large/giant size, vessel branching from the dome, thrombosis of the sac, and previous treatment.

#### 4.3.1. Large/Giant Aneurysm Size and Fusiform Shape

In large/giant and fusiform cases, the decision-making process depends on the availability of proximal and distal control. If these are achievable, the management strategy is progressive clipping reconstruction with temporary clipping. If inflow and outflow control are not achievable, proximal or distal trapping with revascularization should be considered.

Among the eight large/giant aneurysms included in our series, all three cases treated with bypass and trapping were fusiform.

**Figure 1:**
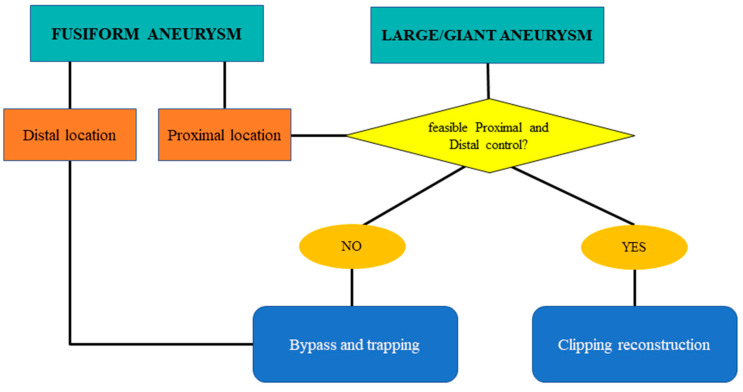
Flow chart for shape management: fusiform and large/giant complex intracranial aneurysms of the MCA.

**Figure 2:**
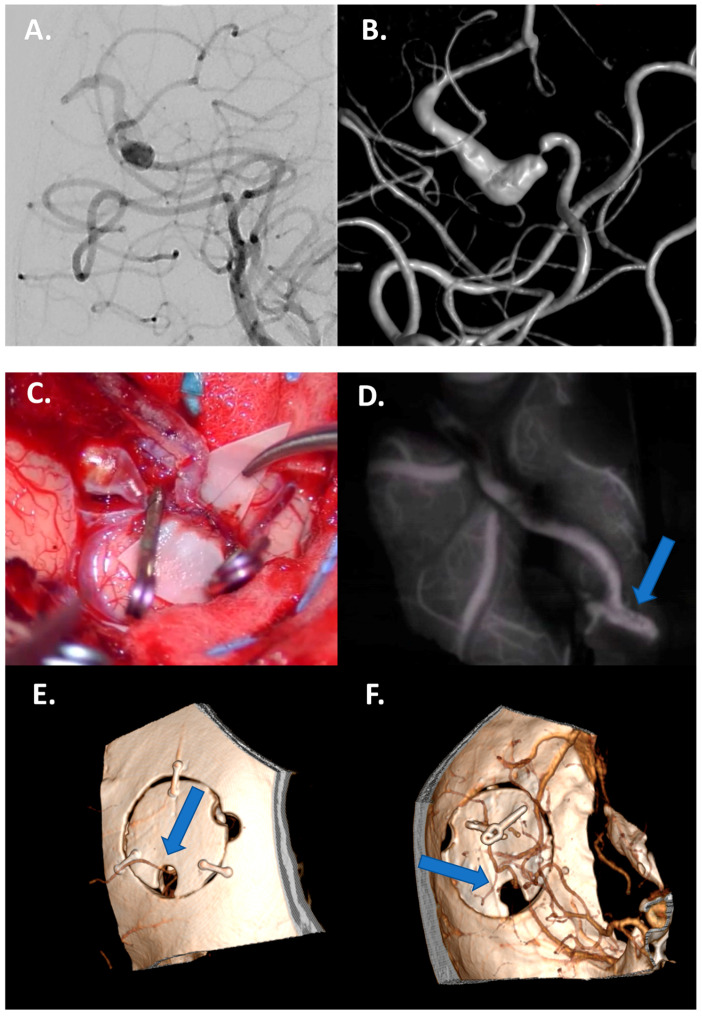
Case 1: Bypass with complete trapping in a large distal fusiform 17 mm aneurysm of M4 in a 24-year-old man. (**A**,**B**) Preoperative DSA with 3D reconstruction documenting M4 tract with a distal stenotic vessel. (**C**,**D**) Intraoperative view of end-to-side superficial temporal artery–MCA bypass anastomosis with complete trapping of the aneurysm by two clips. (**E**,**F**) Postoperative DSA documenting the correct flow replacement with clear view of right superficial temporal artery–MCA bypass through the bone hole (blue arrows).

Fusiform aneurysms generally represent a 360° wall disease; when proximal, they can be “grossly total” reconstructed as a first strategy. Contrarily, distal fusiform aneurysms are unfeasible for reconstruction, and they generally require bypass and trapping (Figure 1 and Figure 2).

Hence, large- or giant-sized aneurysms loom as formidable adversaries, requiring strategic maneuvers to navigate the intricate anatomy of the parent vessels. The magnitude of these lesions underscores the imperative for precision and adaptability in surgical planning, wherein the preservation of critical vasculature assumes crucial significance. The fusiform shape emerges as a hallmark of complexity, heralding challenges in conventional clipping techniques and necessitating innovative approaches for effective exclusion. Its presence mandates meticulous evaluation of the aneurysm’s morphology, delineating pathways for optimal flow restoration while mitigating the risks of rupture or ischemic events.

#### 4.3.2. Vessels Branching from the Dome

**Figure 3:**
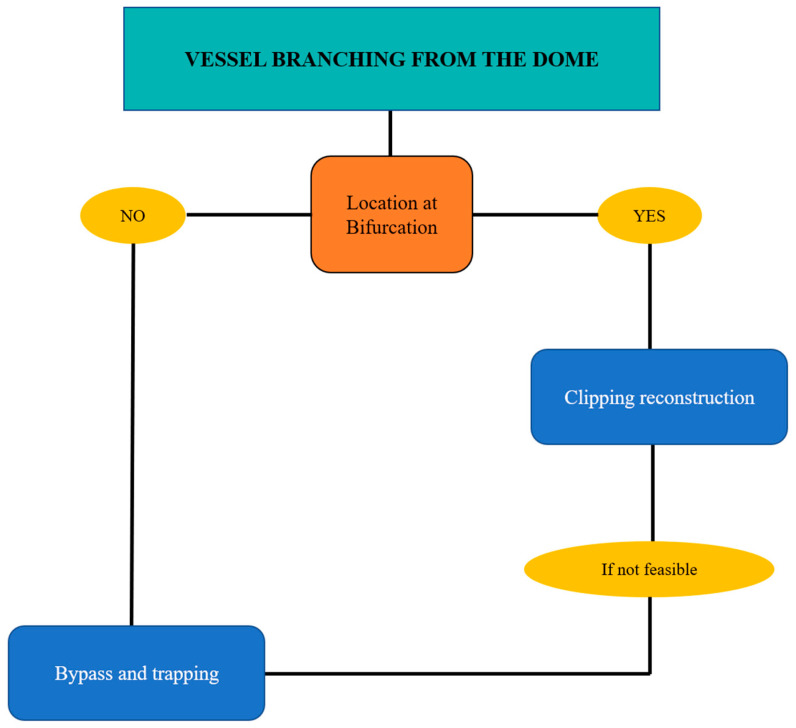
Flow chart for complex aneurysms of the MCA with vessels originating from the dome.

**Figure 4:**
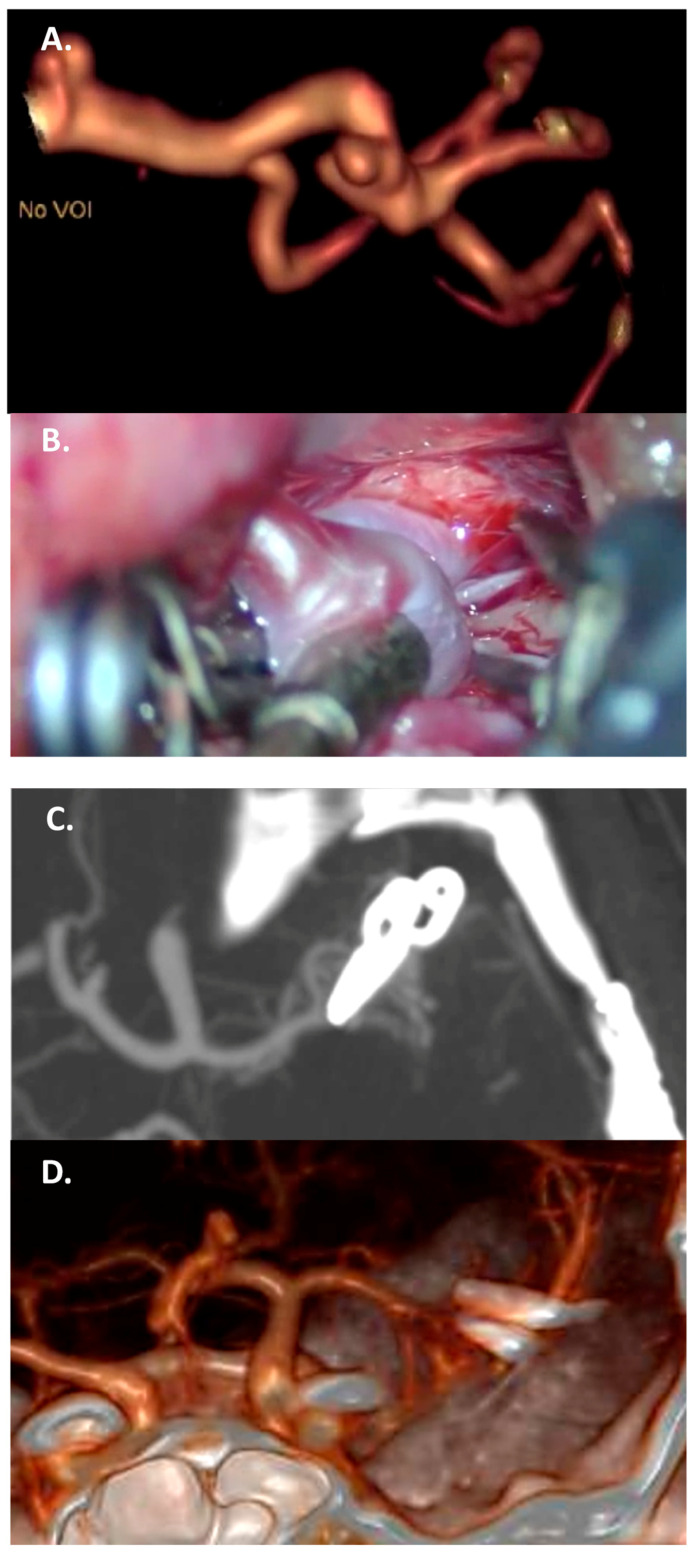
Case 2: complex clipping in a fusiform 7 mm M2 aneurysm with vessel branching from the dome, in a 35-year-old woman. (**A**) Preoperative CTA with a 3D reconstruction. (**B**) Intraoperative view of two tandem clips used to reconstruct the parent vessel. (**C**,**D**) Complete exclusion of the sac, documented by the postoperative CTA, with a 3D reconstruction.

Vessels originating from the dome generally involve aneurysms located at bifurcations. In this case the complete clipping reconstruction is unfeasible; the choice between “gross total” clipping reconstruction with vessel preservation and bypass and trapping depends on size and the morphology of the potential remnant, as well as any history of previous bleeding and the patient’s age. (Figure 3 and Figure 4).

Vessel branching from the dome emerges as a subtle yet pivotal indicator of complexity, accentuating the need for tailored strategies to safeguard vascular integrity.

#### 4.3.3. Thrombosed Aneurysm and/or Previously Treated Aneurysms

**Figure 5:**
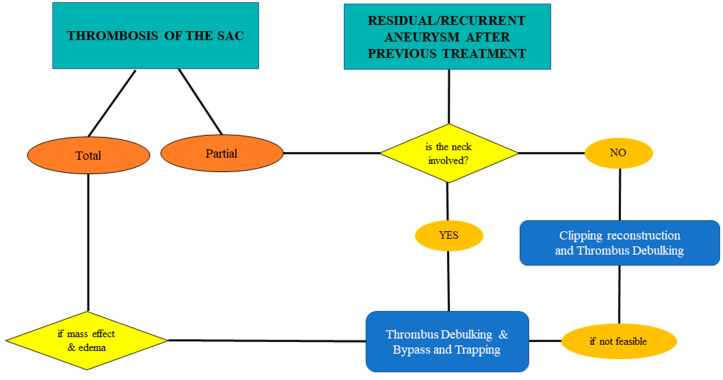
Flow chart for hemodynamic changes of the sac: thrombosed and/or residual/recurrent in previously treated complex aneurysms of the MCA.

**Figure 6:**
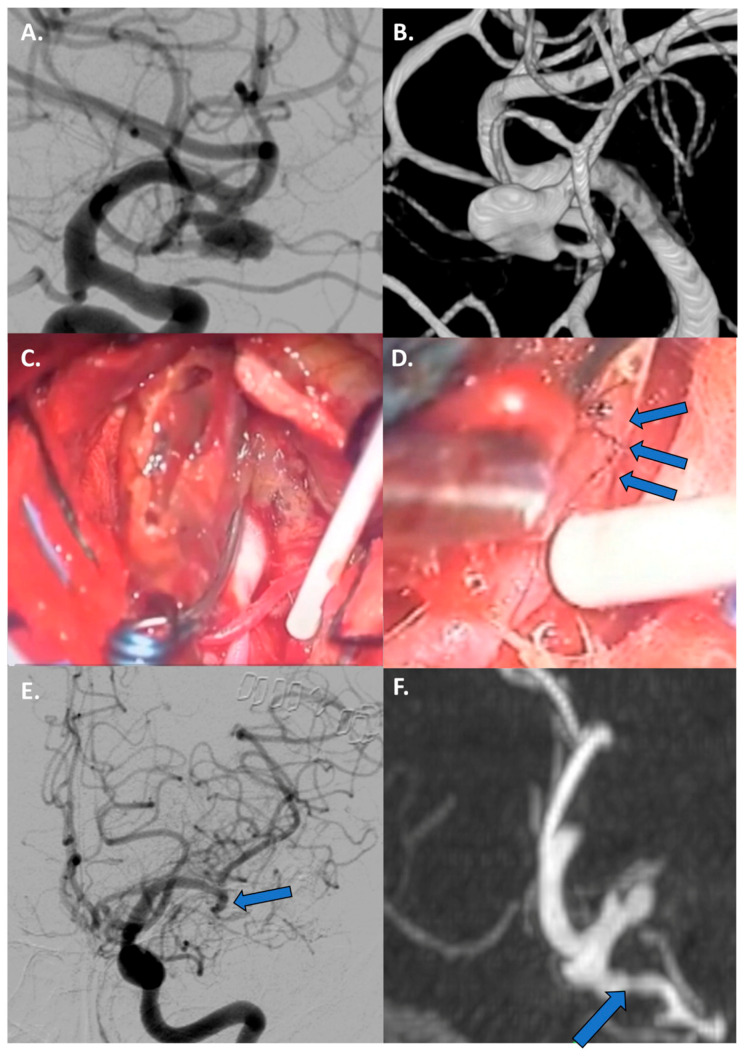
Case 3: Thrombectomy with bypass in a giant, fusiform, and partially thrombosed 21 mm M2 aneurysm carried by a 68-year-old woman with a history of seizures. (**A**,**B**) Preoperative DSA with 3D reconstruction reporting the giant, fusiform, and partially thrombosed aneurysm at left M2. (**C**) Proximal control of M2 performed with a temporary clipping and progressive debulking of intrasaccular thrombus using cavitronic ultrasonic surgical aspirator until visualization of the ostia (blue arrows). (**D**) An intracranial end-to-side bypass between left temporal M3 branch and distal M2 branch. (**E**) DSA confirming bypass flow and flow replacement (blue arrows). (**F**) A 6-month follow-up CTA documenting the complete exclusion of the aneurysm (blue arrows).

**Figure 7:**
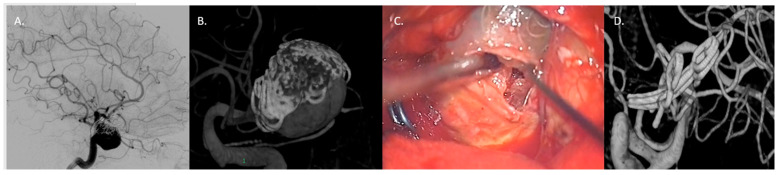
Case 4: complex clipping in previously coiled, giant, partially thrombosed 25 mm right MCA bifurcation aneurysm in a 76-year-old woman. (**A**,**B**) Preoperative presentation with DSA and 3D model of the previously coiled sac with a recurrence at 12 months. (**C**) Surgical clipping with progressive removal of thrombus and coils assisted by ultrasonic aspirator. (**D**) 3D reconstruction DSA confirming the complete exclusion of the sac.

A thrombosed aneurysm’s sac has a potential mass effect on the surrounding parenchyma, with a risk of edema due to the induced inflammatory response [4]. A partially thrombosed aneurysm is unstable, and has a higher risk of bleeding than a completely thrombosed one [37,38,39]. Also, previously treated aneurysms (e.g., coiled) could favor thrombosis or behave as partially thrombosed aneurysms. In these cases, the neck involvement has to be evaluated: If there is no involvement, the best option is clipping reconstruction, considering thrombus debulking. If the thrombus involves the neck, thrombus debulking with bypass and trapping is mandatory (considering the high risk of parent vessel thromboembolism). For completely thrombosed aneurysms, treatment has to be considered in symptomatic cases (given, e.g., mass effect and edema) and the choice is the same as for partially thrombosed cases with neck involvement (Figure 5, Figure 6 and Figure 7).

Thrombosis represents a threat for hemodynamics due to the risk of progression through the neck or embolism and necessitates real-time adaptations of surgical technique to ensure optimal patient outcomes. Furthermore, the thrombosed aneurysm could behave as a tumoral mass, inducing edema in the surrounding parenchyma. Similarly, previous surgical or endovascular treatments may cause inflammatory reactions, increasing the risk of the sac and parent vessels’ manipulation.

## 5. Limitations

This study has some limitations. First, the small number of patients involved; multicentric collaboration might produce further results and stronger correlation analysis concerning the treatment of complex unruptured MCA aneurysms. Furthermore, our analysis is focused on the surgical point of view; it lacks comparisons with the endovascular strategies. Finally, the proposed flow charts are qualitative, with no external validation. We assumed the reliability of the treatment strategies on the basis of surgical outcomes in our center, which are superimposable to the population of non-complex MCA aneurysms.

## 6. Conclusions

The surgical treatment of unruptured complex aneurysms of the MCA remains a challenge for the neurosurgeon. The identification of major criteria such as fusiform shape, large/giant size, presence of thrombosis, branch vessels originating from the sac, and previous treatments, as well as their combination in the specific aneurysm case, have to be addressed in surgical management. We believe that the decisional algorithm of management should be routinely assessed on the basis of a simultaneous evaluation of the complexity profile of the aneurysm and patient characteristics. The management of complex MCA aneurysms benefits from the use of an algorithmic approach based on complexity profiles to effectively guide treatment decisions. Our institutional series highlights the relevance of major complexity criteria which have a specific interplay in shaping the choice of surgical strategy. In modern healthcare settings, having a diverse portfolio of surgical techniques, including trapping and bypass procedures, is crucial. The neurosurgical team must be skilled and trained to perform extracranial–intracranial or intracranial–intracranial bypasses in high volume at a specialized neurovascular center. However, complex clipping reconstruction should represent the first choice in the treatment of complex MCA aneurysms, assuming the bypass revascularization to be a rescue strategy.

## 7. Patients

All patients expressed their written consent to the treatment of their personal data for scientific purposes, contextually with the informed consent for surgery.

## Figures and Tables

**Table 1:** Major and minor complexity features in our series of complex unruptured MCA aneurysms.

Complexity Features	N (%)
***Major***	
Fusiform shape	8 (57%)
Large size (10–25 mm)	5 (35%)
Giant size (>25 mm)	3 (21%)
Blister or <3 mm size	0 (0%)
Vessel branching from the dome	7 (50%)
Thrombosis	5 (35%)
Previous treatment	2 (14%)
***Minor***	
Wide neck	13 (92%)
Proximal vessel tortuosity/stenosis	4 (28%)
Calcification	2 (14%)

**Table 2:** Study population, demographical data, aneurysm size and location, clinical presentation, surgical treatment, and outcome for complex unruptured MCA aneurysms in our series.

Characteristics	N* (%)
***Mean basal age (± SD) yo***	54.85 ± 16.18
***Female/male ratio***	9:5
***Mean aneurysm size (± SD) mm***	14.36 ± 8.7
***Location of the aneurysm in MCA***	
M1	3 (21%)
First bifurcation	3 (21%)
M2	5 (35%)
Second bifurcation	1 (7%)
M3	2 (14%)
***Preoperative mRS***	
0–2	11 (78%)
3–4	3 (22%)
***Clinical presentation***	
None	5 (36%)
Headache	10 (71%)
Vertigo	2 (14%)
Seizure	3 (21%)
Hemiparesis	1 (7%)
***Risk factors***	
Hypertension	10 (71%)
Smoking	5 (35%)
Alcohol	1 (7%)
Drug abuse	0 (0%)
Familiarity for SAH	0 (0%)
Diabetes	2 (14%)
Vasculopathy	3 (21%)
***Surgical treatment***	
Complex clipping reconstruction	9 (64%)
Trapping and bypass	5 (36%)
***Postoperative mRS***	
0–2	13 (93%)
3–5	1 (7%)
***Complication***	3 (21%)
Neurological	2 (14%)
Systemic	1 (7%)

M1: first tract of middle cerebral artery; M2: second tract of middle cerebral artery; MCA: middle cerebral artery; mm: millimeters; mRS: modified Rankin scale; N*: number; SAH: subarachnoid hemorrhage; SD: standard deviation; yo: years old.

**Table 3:** Comparison between our series and the entire institutional series of MCA surgically treated aneurysms.

	Complex Unruptured MCA (14)	Non-Complex Unruptured MCA (188)	*p* Value
**Mean age**	54.85 ± 16.18	59.41 ± 10.48	0.39 *
**F:M ratio**	9:5	27:7	0.18
**Mean Size (mm)**	14.36 ± 8.7	5.97 ± 2.80	0.00001 *
**Preoperative mRS (0–2)**	11 (78%)	174 (100%)	0.00003
**Preoperative mRS (3–6)**	3 (22%)	0	
**Postoperative mRS (0–2)**	13 (93%)	172 (99%)	0.208
**Postoperative mRS (3–6)**	1 (7%)	2 (1%)	
**Complications**	2 (14%)	24 (14%)	0.34
**Complete exclusion** **Incomplete exclusion**	12 (86%)	145 (83%)	1
	2 (14%)	29 (17%)	

* Mann–Whitney *U* test; mRS: modified Rankin scale; F: female; M: male.

**Table 4:** Locations, complexity features, and surgical strategies for complex MCA aneurysms in our series.

Patient	MCA Location	“Major” Complexity Criteria						Surgical Strategy
		*Fusiform*	*Large*	*Giant*	*Vessel Branching*	*Thrombosis*	*Previous Treatment*	
* #1. *	Bifurcation			√		√	√	CR
* #2. *	M1	√			√			CR
* #3. *	M3–M4	√	√					BT
* #4. *	M1			√				BT
* #5. *	M1	√		√	√		√	CR
* #6. *	M2	√			√	√		BT
* #7. *	Bifurcation		√					CR
* #8. *	M2		√			√		CR
* #9. *	M3	√	√			√		BT
* #10. *	M2	√			√			BT
* #11. *	M2		√					CR
* #12. *	Bifurcation				√			CR
* #13. *	M3	√			√	√		CR
* #14. *	M2	√			√			CR

BT: bypass and trapping; CR: clipping reconstruction; M1: first tract of middle cerebral artery; M2: second tract of middle cerebral artery; M3: third tract of middle cerebral artery; M4: fourth tract of middle cerebral artery; MCA: middle cerebral artery.

## Data Availability

The dataset that supports the findings of this study is available from the corresponding author, A.A., upon reasonable request.
